# Short-term sleep fragmentation enhances anxiety-related behavior: The role of hormonal alterations

**DOI:** 10.1371/journal.pone.0218920

**Published:** 2019-07-03

**Authors:** Zeljko Grubac, Nikola Sutulovic, Anida Ademovic, Milica Velimirovic, Aleksandra Rasic-Markovic, Djuro Macut, Natasa Petronijevic, Olivera Stanojlovic, Dragan Hrncic

**Affiliations:** 1 Laboratory of Neurophysiology, Institute of Medical Physiology “Richard Burian”, Faculty of Medicine, University of Belgrade, Belgrade, Serbia; 2 Institute of Clinical and Medical Biochemistry, Faculty of Medicine, University of Belgrade, Belgrade, Serbia; 3 Clinic of Endocrinology, Diabetes and Metabolic Disease, CCS, Faculty of Medicine, University of Belgrade, Belgrade, Serbia; Technion Israel Institute of Technology, ISRAEL

## Abstract

**Introduction:**

The neuroendocrine background of acute sleep fragmentation in obstructive sleep apnea and sleep fragmentation involvement in psychiatric comorbidities, common in these patients, are still largely unknown. The aim of this study was to determine the effects of short-term experimental sleep fragmentation on anxiety -like behavior and hormonal status in rats.

**Methods:**

Male rats were adapted to treadmill (ON and OFF mode with belt speed set on 0.02m/s and 0.00m/s) and randomized to: 1) treadmill control (TC, only OFF mode); 2) motion, activity control (AC, 10min ON and 30min OFF mode) and 3) sleep fragmentation (SF, 30s ON and 90s OFF mode) group. Six hours later, the animals were tested in the open field, elevated plus maze and light/dark test (n = 8/group). Testosterone, estradiol, progesterone and corticosterone were determined in separate animal cohort immediately upon sleep fragmentation (n = 6/group).

**Results:**

SF rats showed decreased rearings number, decreased time spent in the central area and increased thigmotaxic index compared to TC and AC rats in the open field test. Similarly, increased anxiety upon sleep fragmentation was observed in the elevated plus maze and the light/dark test. Significantly lower testosterone, estradiol and progesterone levels were determined in SF in comparison to AC and TC groups, while there was no significant difference in the levels of corticosterone.

**Conclusion:**

Short term sleep fragmentation enhances anxiety-related behavior in rats, which could be partly mediated by the observed hormonal changes presented in the current study in form of testosterone, estradiol and progesterone depletion.

## Introduction

Sleep is a physiological process of diminished vigilance without which human life is impossible. The consequences of inadequate sleep quality and quantity, i.e. sleep deprivation and fragmentation are nowadays being increasingly recognized as significant health problem. Actually, we are a sleep-deprived society which sleeps 6.8h on average as opposed to 9h per day a century ago [1]. The 24/7 economy and its subsequent impact on sleep patterns test the limits of our bodies to maintain physical, mental and hormonal equilibrium [2]. The beneficial effects of sleep can only be achieved if its duration and architecture remain uninterrupted. Namely, inadequacy of the quantity and the architecture of sleep are associated with numerous diseases ranging from cardiovascular to neurological (e.g. epilepsy) and psychiatric disorders [3–5]. Recent evidence suggested a bidirectional relationship between psychiatric disorders and sleep disturbances, in which they reinforce each other and share common and overlapping mechanisms [6,7]. Altered duration and quality of sleep symptoms are established diagnostic criteria for posttraumatic stress disorder (PTSD) and generalized anxiety disorder [8]. Actually, sleep disturbance may be the very first presenting symptom in these disorders [9,10].

Sleep deprivation and sleep fragmentation are two major types of alteration of physiological sleep pattern. Sleep deprivation is characterized by sleep loss, restricted sleep duration, or REM sleep exclusion. Contrary to sleep deprivation, total sleep time is insignificantly diminished in patients experiencing sleep fragmentation. Instead, sleep fragmentation consists of frequent, brief arousals followed by a rapid sleep onset, thereby modulating regular sleep architecture [11,12].

Sleep fragmentation may occur in patients with various psychiatric, medical and respiratory problems, especially in patients who suffer from obstructive sleep apnea (OSA) [13]. Daily somnolence, lack of attention and reduced cognitive abilities are common consequences of sleep fragmentation [14]. It has been reported that almost 17% of military veterans with OSA also had an anxiety disorder [15]. The comorbidity between OSA and anxiety is striking. However, the majority of work conducted on this association has specifically focused on the relation between OSA and PTSD explaining it by OSA-induced hypercapnia [16,17]. OSA is characterized by hypercapnia and sleep fragmentation [17]. It is not clear how sleep fragmentation in OSA contributes to psychiatric comorbidities.

Studies on humans and animals have shown that sleep alterations, in form of sleep deprivation and sleep restriction, are often associated with mild, temporary increase in the activity of the autonomic sympathoadrenal system and the hypothalamic-pituitary-adrenal (HPA) axis, as well as the effects in hormonal dysregulations in general [2]. These dysregulations could be monitored via plasma corticosterone, testosterone, progesterone and estradiol levels. However, the increased sympathetic activation is more related to disturbances in sleep architecture than to sleep deprivation [18]. On the other hand, current knowledge regarding other neuroendocrine background of acute sleep fragmentation is limited.

Animal models have been and continue to be an indispensable research strategy for understanding the pathogenesis of psychiatric disorders. It has been generally accepted that exploratory behavior is sensitive to alterations in anxiety levels [19]. The ethological test armamentaria for exploring anxiety-related behavior usually include open field test, light dark test and elevated plus maze test.

Therefore, the aim of this study was to determine the acute effects of short-term sleep fragmentation similar to those seen in patients with OSA, on the exploratory behavior in male rats by using a battery of behavioral tests to gain clear insight into the effects of acute sleep fragmentation on anxiety levels. Plasma corticosterone, testosterone, progesterone and estradiol levels were measured to determine if a HPA axis response could mediate the behavioral changes observed after the treadmill-induced sleep fragmentation.

## Materials and methods

### Ethical statement

All experimental procedures were in full compliance with the European Council Directive (2010/63/EU) and approved by the Ethical Committee of the Faculty of Medicine, University of Belgrade.

### Animals and housing

The experiments were conducted on adult male Wistar albino rats (2 months old at the arrival) obtained from the Military Medical Academy breeding laboratory (Belgrade, Serbia). The animals were housed individually in Plexiglas cages (55x35x30 cm) with free access to food and water during the entire experiment. They were kept under controlled ambient conditions (22–24°C, 50±5% relative humidity, 12:12h light/dark cycle (with light switched on from 8:00h to 20:00h). The acclimatization period to laboratory conditions lasted for one full week, and all animals were used only once during the experiment.

### Experimental design and sleep fragmentation method

Sleep fragmentation was achieved through an already established and widely used method of sleep fragmentation reported in literature (e.g. [17, 20–22]) based on the use of treadmill apparatus for small animals, which included control group, additional control locomotor activity group and study group with sleep fragmentation. We used the protocol described in details in McKenna et al. [17].

The treadmill apparatus (*NeuroSciLaBG-Treadmill*, *Elunit*, Serbia) used in this study consists of a conveyor belt with motion programmed in advance with the customized control program, as reported in our previous study [3]. The treadmill activity was defined by ON mode (belt moving alternatively forwards or backwards) at the speed of 0.02 m/s and OFF mode at the speed of 0.00 m/s. We applied the method during the first 6h of light phase of the light/dark cycle.

All animals were adapted to the treadmill two days prior to the experiment in 1-hour sessions, in which the ON and OFF mode were alternately rotated in 5 min cycles (5 min ON: 5 min OFF, 1h). Intervals of sleep fragmentation were set to match the sleep of patients with obstructive sleep apnea. Every hour had 30 equally divided sections corresponding to fragments of 2 min each, with alternate rotations of both ON and OFF treadmill activity. Therefore, the treadmill was programmed to alternately work for 30s ON and 90s OFF over a period of 6h in the sleep fragmentation group (Sleep fragmentation group, 30 sleep interruptions per hour, SF; n = 8).

The corresponding activity control group was included in the study in order to control the confounding effects of movements itself. In this group, the total quantity of motion was equal to SF group, but with longer period of undisturbed sleep (1.5 interruptions per hour *vs*. 30 interruptions per hour in SF group), which is similar to other studies [23,24]. Hence, the treadmill in this group was programed to operating mode of 10min ON and 30min OFF (Activity control group, 1.5 sleep interruptions per hour, AC; n = 8).

The treadmill control consisted of rats staying in the treadmill apparatus at moving speed of 0 m/s and conditions equivalent to those in the cages (Treadmill control group, 0 sleep interruptions per hour, TC; n = 8).

After 6h rats from SF, AC and TC groups were subjected to ethological tests to assess the anxiety-related behavior. These tests included standard battery of tests consisting of open field, light/dark and elevated plus maze test, to which the rats have been always subjected in the same order.

Additional cohort of animals (n = 6 per group) underwent the same treadmill regimes and were sacrificed for biochemical serum hormone levels determination.

**Fig 1:**
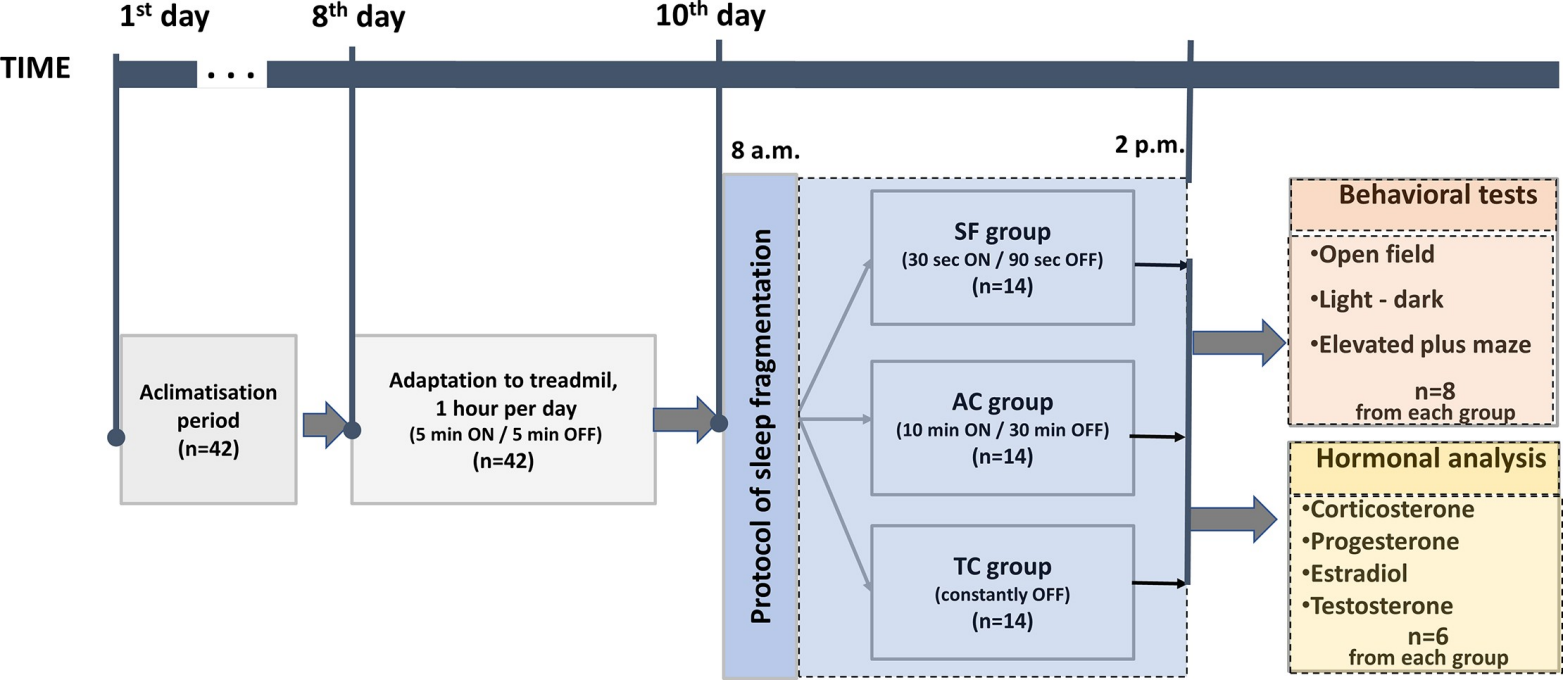
Schematic illustration of experimental design time flow. Animals (n = 42) were acclimatized to the laboratory conditions (7 days). Adaptation to the treadmill was performed in sessions of 1h (5 min ON and 5 min OFF working regime, treadmill belt speed ON = 0.02 m/s, OFF = 0m/s) during the next 3 days. Afterwards, the sleep fragmentation protocol has been applied and animals were divided into three groups (n = 14 in each group) and subjected to one of the three protocols in the treadmill during 6h of the light period: 1) sleep fragmentation (30s OFF: 90 s ON regime, 30 sleep interruptions per hour, SF group), 2) activity control group (10min ON: 30 min OFF regime, 1.5 sleep interruptions per hour, AC group), 3) treadmill control group (constantly OFF regime, 0 sleep interruptions per hour, TC group). Upon completion of sleep fragmentation protocols one cohort of animals (n = 8 from each group) has been subjected to in vivo ethological test of anxiety-like behavior (open filed, light—dart and elevated plus maze test), while another cohort of animals (n = 6 from each group) was sacrificed for blood sampling and further in vitro ELISA determination of hormonal levels (testosterone, progesterone, estradiol, corticosterone). For further details see the Materials and Methods section.

The study design time-flow chart is presented in Fig 1.

### Open field test

Rat open field behavior was monitored by an automated system fully equipped with infrared sensors (Experimetria Ltd., Budapest, Hungary) and its accompanied software program (Conducta 1.0), as described in details in our previous study [25]. Briefly, this system registered the horizontal and vertical activity of the animals gently placed in the center of the sound-attenuated area (48 cm × 48 cm) with red lighting of 12 lx, which was surrounded by black walls (height 40 cm). The system recorded the distance and time of ambulatory movement, as well as the number of rearings during rat exploration of the novel environment. Recording sessions lasted for 15 min. Subsequently, the whole area was divided by the software into 16 squares of which the 4 middle squares are marked as the central area. The time that an animal spent in the central area was measured. Thigmotaxic index was calculated as a ratio between the distance of rat ambulatory movements in the peripheral zones and the total distance of ambulatory movements (%).

### Light-dark test

For the light-dark test, a rat was placed in the center of the light compartment (27×27×27 cm, all surfaces in white) which was connected with the dark compartment (27×18×27 cm, all surfaces painted in black) by a square aperture (8×8 cm, Elunit, Belgrade, Serbia). Rat activity was video monitored during the following 5 min and analyzed offline by an investigator blinded to the treatment. The time that an animal spent in the light compartment of light/dark test, as well as the number of transitions from light to dark compartment were measured as indicators of anxiety-related behavior.

### Elevated plus maze test

The elevated plus maze apparatus consisted of two open arms (50x10 cm) and two enclosed arms (50x10x40 cm) arranged in such a way that two pairs of identical arms were opposite to each other. Arms emerged from a central platform (10x10 cm), and the entire apparatus was raised to a height of 50 cm above floor level. At the beginning of the test, the rat was placed on the central platform facing an open arm. After each 5-min test, the maze was carefully cleaned up to avoid any olfactory trace of previous animal. Anxiety-related behavior was assessed through the following behavioral parameters forming output variables of this test: time in open arms and overall number of transitions between opened and closed arms. These parameters are inversely related to the anxiety level in rodents.

### Biochemical hormone level determination

Separate cohort of animals from SF, AC and TC groups (n = 6) were sacrificed by decapitation immediately upon completion of one of the assigned treadmill regimes. Blood samples were collected and centrifuged at 1575 *g* for 10 min. Serums were collected and frozen at -80°C until assayed for hormone levels. Testosterone, estradiol, progesterone and corticosterone were determined in sampled serums by commercially available kits using the method of enzyme immunoassay (ELISA Kit, Cusabio Technology LCC) according to manufacturer’s instructions. Minimum detectable concentrations were: 0.06 ng/ml for testosterone (detection range: 0.13–25.6 ng/ml), 0.2 ng/ml for progesterone (1–30 ng/ml), less than 40 pg/ml for estradiol (40–1400 pg/ml) and 0.1 ng/ml for corticosterone (0.2–40 ng/ml). Duplicate serum aliquots for all hormone analyses were used. These assays are routinely performed in our biochemical laboratory.

### Data analysis and statistics

The Kolmogorov-Smirnov test was used to test the normal distribution of values. The analyzed parameters obtained from the open field, light/dark and elevated plus maze test, as well as the hormone level values showed normal distribution. Therefore, the results were expressed as a means ± standard error (SE) for all parameters. The statistical difference between the groups was estimated by using One-Way ANOVA with Fisher's LSD post hoc test. The exception was the number of transitions in the light/dark test for which the normal distribution was not estimated. Results on this parameter were expressed as medians with 25^th^– 75^th^ percentiles and statistical difference between the groups was estimated by Kruskall-Wallis One-Way ANOVA with Mann-Whitney U test. The criteria for the significance of statistical differences were *p<0.05, **p<0.01

## Results

**Fig 2:**
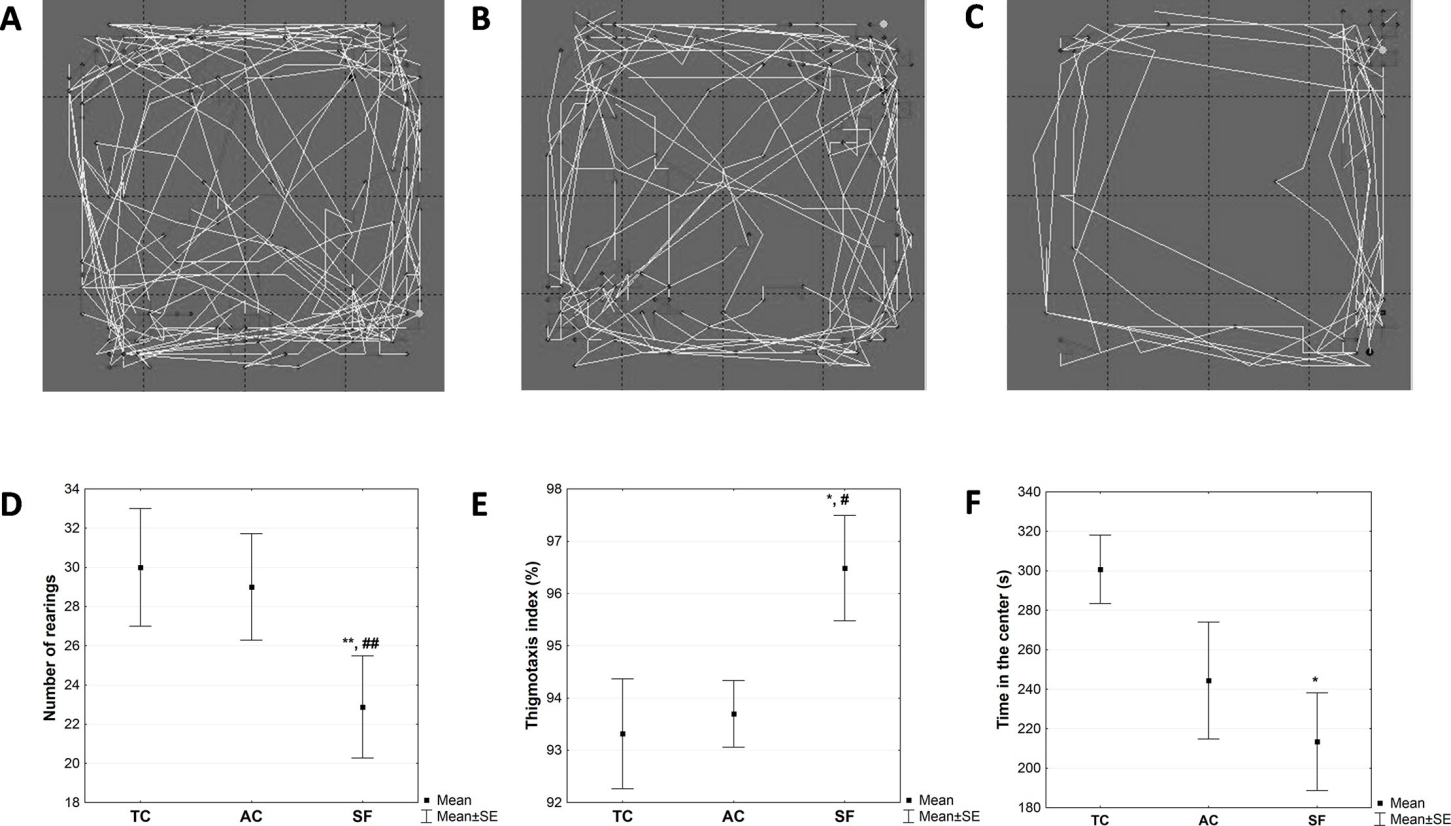
**Representative traces of locomotor activity of animals in the open field test in the (A) treadmill control, (B) activity control and (C) sleep fragmentation groups. Number of rearings (D), thigmotaxis index (E) and time spent in the central area (F) in the open field test registered in animals upon sleep fragmentation method.** The number of rearings was calculated as the number of times rat has propped on the hind legs. The index of thigmotaxis was defined as a ratio between the distance of ambulatory movement a rat made in the peripheral areas and the total distance of ambulatory movements in the open field test. Values are mean ± SEM. The statistical difference between groups was estimated by using One-Way ANOVA with Fisher’s LSD post hoc test (*p < 0.05, **p < 0.01 vs. TC; ^#^p < 0.05, ^##^p < 0.01 vs. AC). For further details see the caption to Fig 1.

Analysis of the locomotor activity of animals in the open field test at the end of the appropriate treadmill regime showed different behavioral patterns in the TC, AC and SF groups, as shown in representative traces of ambulatory movements in the arena used for this test (Fig 2A–2C).

Further quantitative analysis of the anxiety-related output variables in the open field test showed no differences between the two control groups (TC *vs*. AC group) in any of the analyzed parameters (p>0.05, Fig 2D–2F). On the other hand, differences were revealed between the rats that underwent sleep fragmentation regime (SF group) and those that underwent control regimes (TC and AC groups). According to One-Way ANOVA with LSD post hoc test, the SF group of rats showed significant decrease in the number of rearing, an indicator of vertical activity, compared to both TC (p< 0.01) and AC group (p<0.01, Fig 2D). The index of thigmotaxis was significantly higher in the SF group of rats in comparison to both TC (p< 0.05) and AC group (p< 0.05, Fig 2E). As for the time spent in the central area of the open field test, it has been shown that rats from the SF group spent significantly less time in the central area compared to the rats from the TC group (p< 0.05, Fig 2F).

**Fig 3:**
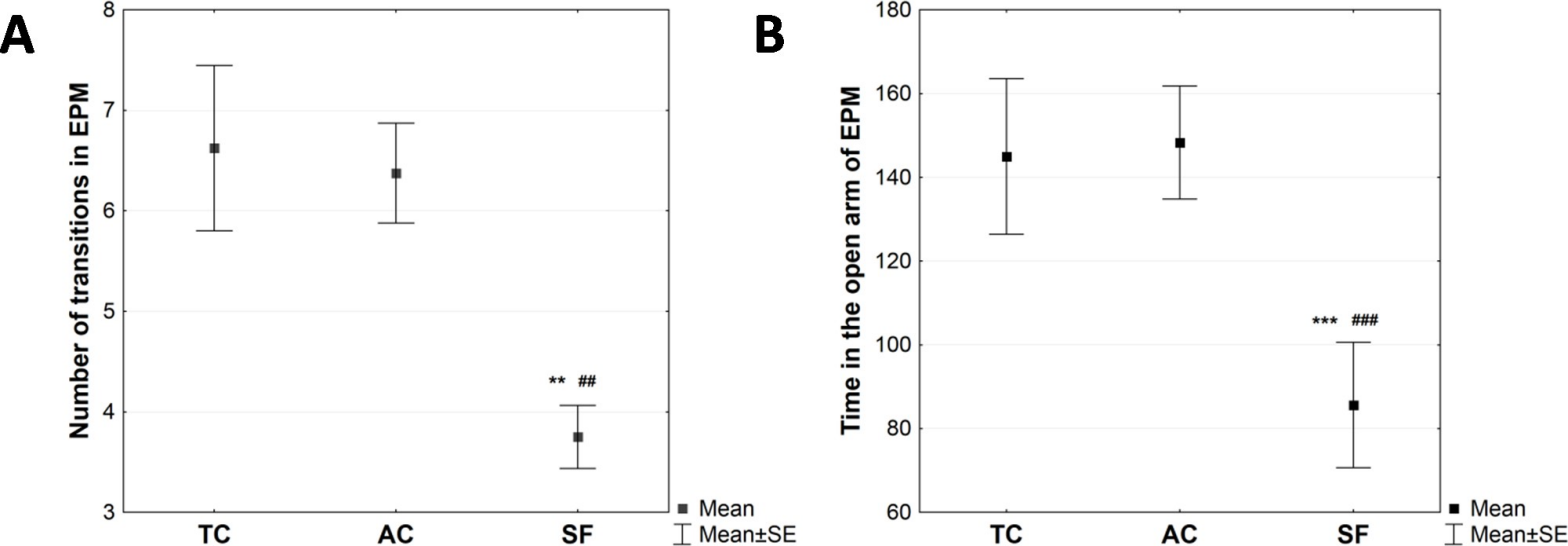
**The number of transitions between the opened and closed arm (A) and the time spent in the open arm (B) in elevated plus maze test (EPM) observed in animals from treadmill control (TC), activity control (AC) and sleep fragmentation (SF) group upon sleep fragmentation method.** Values are mean ± SEM. The statistical difference between the groups was estimated by using One-Way ANOVA with Fisher’s LSD post hoc test (**p < 0.01, ***p = 0.001 vs. TC, ^##^p < 0.01, ^###^p = 0.001 vs. AC). For further details see the caption to Fig 1.

The results of elevated plus maze test are shown in Fig 3. No differences between the TC and AC group in any of the analyzed parameters derived from elevated plus maze test were detected (p>0.05, Fig 3A and 3B). The sleep fragmentation protocol significantly decreased the time animals spent in the open arms during the test compared to both control protocols (SF *vs*. AC and SF *vs*. TC, p< 0.01, Fig 3B). In addition, the number of open/closed arms transitions was significantly decreased in the SF compared to the AC and TC groups (p< 0.001, Fig 3A).

**Fig 4:**
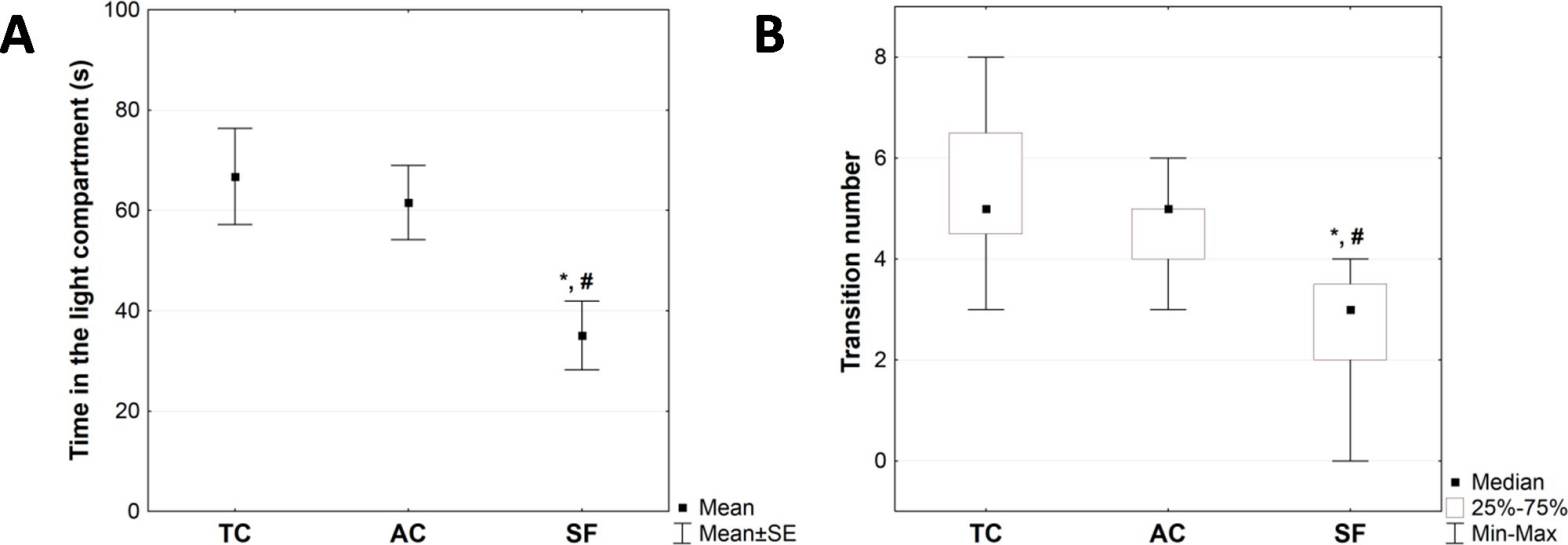
**The time in the light compartment (A) and the number of transitions between the light and the dark compartment (B) in light/dark test observed in experimental and control groups.** The statistical difference between groups in the time in the light compartment was estimated by using One-Way ANOVA with Fisher’s LSD post hoc test and in the number of transitions by Kruskall Wallis ANOVA and Mann Whitney U test (*p < 0.05 vs. TC, #p < 0.05 vs. AC). For further details see caption to Fig 1.

Ethological findings derived from the light/dark test are presented in Fig 4. The time the animals spent in the light compartment was significantly shorter in the SF group compared to the AC and TC groups (p<0.05, Fig 4A). The same applies to the number of light/dark compartment transitions (SF *vs*. AC and SF *vs*. TC, p<0.05, Fig 4B). These light/dark test parameters were not significantly different between the TC and AC group (p>0.05, Fig 4A and 4B).

**Fig 5:**
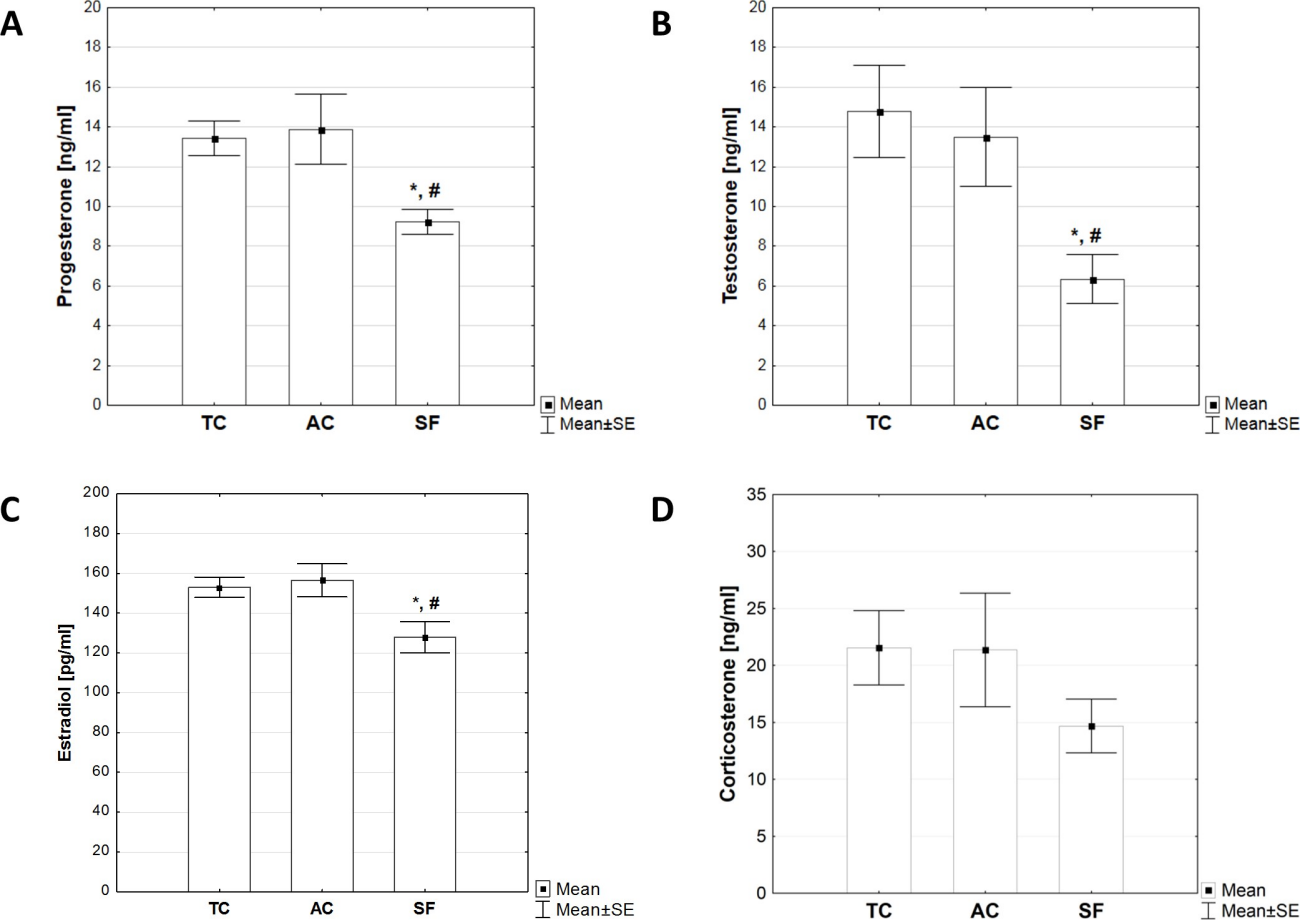
**Serum levels of progesterone (A), testosterone (B), estradiol (C) and corticosterone (D) observed in animals from treadmill control (TC), activity control (AC) and sleep fragmentation (SF) group immediately after the experiment.** Hormone serum concentrations were determined in separate cohort of animals immediately upon sleep fragmentation method (n = 6 per group) using ELISA method. Values are mean ± SEM. The statistical difference between groups was estimated by using One-Way ANOVA with Fisher’s LSD post hoc test (*p < 0.05 vs. TC, #p < 0.05 vs. AC). For further details see caption to Fig 1.

The results of serum hormone level determination by ELISA kits reveled no significant differences in the levels of testosterone, estradiol, progesterone and corticosterone between the TC and AC group (p>0.05, Fig 5). On the other hand, animals subjected to sleep fragmentation regime in the treadmill (SF group) had significantly lower serum levels of testosterone, estradiol and progesterone in comparison to both AC and TC control groups (p< 0.05, Fig 5A–5C). Unlike those hormones, there was no significant difference in the levels of corticosterone between SF and both AC and TC groups (p >0.05, Fig 5D).

## Discussion

Sleep fragmentation in this study was experimentally modeled to disrupt sleep with the frequency present in the patients with OSA. The results of all three ethological tests (open filed, elevated plus maze and light/dark test) applied in our study are consistent and corroborate an increased anxiety level upon sleep fragmentation according to standard interpretation [26–28]. Namely, these tests of anxiety-linked behavior strongly imply reduction of exploratory behavior after being subjected to the acute sleep fragmentation regime. The decreased time spent in the central area, as well as the number of rearings, the significant increase in the thigmotaxic index and the prolonged time spent in the dark field indicate that the animals are less interested in exploring the unknown environment. This behavioral pattern matches the image of the anxiety-linked behavior [26]. Moreover, the increase in index of thigmotaxis, the most sensitive sign, means that the animals moved more in the corners and areas along the walls of the open field arena when analyzed in relation to the total distance of ambulatory movement. This is in accordance with the decrease in the time spent in the central area, also indicating less interest in exploring the unknown environment. On the other hand, decrease in the number of rearing merely adds up on the decrement in exploratory activity, just in this case it refers to the vertical activity.

The results of the previous studies regarding the link between sleep fragmentation and anxiety are not homogenous. Namely, sleep fragmentation increased grooming, but it had no effect on stereotyped behaviors, locomotion, or the elevated plus maze test according to the results of Tufik et al. [19]. On the other hand, it has been also shown that sleep fragmentation significantly decreased rearing behavior, indicator of vertical activity associated with exploratory activity (Andersen et al., 2005). In addition, Suchecki et al. [29] showed that rats had reduced locomotor activity and augmented anxiety-like behavior upon sleep fragmentation achieved by single platform. Moreover, lethal outcomes after a chronic exposure to sleep fragmentation were reported in the experimental conditions [19]. The possible underlying explanation for the diverse spectrum of results can be found in the interpretation of the results or the method used to induce the sleep disruption.

Manipulation of sleep fragmentation in our study has been designed according to an already established and widely used method of sleep fragmentation reported in literature, which includes control group, additional control locomotor activity group and study group with sleep fragmentation, i.e. TC, AC and SF group in our study respectively ([20] and others stated in the Methods section). The protocol duration (6h, 1day or several days), the rodent animal strain, as well as the protocol of movement device (treadmill, rotating wheal or drum) are variable. We used those which are the most similar to the ones used by McKenna et al. [17]. The majority of these studies used the same frequency to disrupt sleep (30 interruptions per hour) to mimic those in patients with severe OSA.

According to previous studies in which polysomnographic recordings were performed [17], animals exposed to 6h of—sleep fragmentation protocol used herein significantly increased the total percent of time spent awake (from 29.2% baseline to 65.0%). Further, the percentage of both NREM and REM sleep was decreased in these animals and the same applies to average NREM episode duration. All aforementioned findings support the fact that these animals had fragmented and ragged sleep. Additional tests in recovery phase also confirm these findings. Taking into account these results together with our previous experience with this methodology [3], we did not repeat sleep recordings in the current study in order to keep animal number as low as possible for ethical reasons.

If we take a look at the design of TC, AC and SF groups (0, 1.5 and 30 sleep interruptions per hour respectively) we can observe that the AC group has negligible sleep interruptions. The protocol for the AC group was introduced to control the confounding effects from movement, similarly to other studies [23]. It offers the same walking distance, but with longer period of undisturbed sleep (1.5 interruptions per hour *vs*. 30 interruptions per hour in SF group) [24]. This could explain the lack of significant differences between the TC and the AC group (0 *vs* 1.5 sleep interruptions per hour) in majority of output variables observed in this study.

Clinical studies highlight an increased anxiety-like behavior upon sleep fragmentation, which we also proved experimentally in the current study. According to reported cases, there is an increase in self-reported anxiety, frustration, loss of interest and motivation in patients with obstructive sleep apnea [30–32]. Furthermore, a higher level of traffic accidents has been reported in these patients, due to lack of concentration and problems with cognition [33]. Moreover, when analyzing effects of sleep disruption on mood fluctuations and anxiety, we should bear in mind the bidirectional link between these entities and that differentiation between cause and consequence is complex. Apart from sleep fragmentation, some studies suggested that sleep deprivation can be used as a model of mania in rats due to its locomotor “hyperactivity” [7,31].

The first signs of behavioral alterations upon sleep fragmentation are supposed to manifest on the emotional level [34, 35]. However, sleep disruption in the long run ultimately changes the fundamental properties of neuroendocrine stress systems thus affecting anxiety levels [36]. In this study, testosterone, estradiol and progesterone levels were significantly lower in the group that underwent sleep fragmentation compared to both controls (p<0.05, SF *vs*. AC and SF *vs*. TC), which suggests deregulation of HPA axis. At the same time, there were no differences in corticosterone levels upon sleep fragmentation.

There is still a lack of results in the area of hormonal changes induced by sleep fragmentation. Increased activity of HPA axis by acute sleep deprivation, according to Han et al. [37], is assumed to be the consequence of mental and/or physical stress rather than insufficiency of sleep itself. At least part of the acute effects is a result of mechanisms related to corticotropin releasing hormone (CRH). CRH neurons which form a feed-forward system with brain stem noradrenergic (NA) system (*i*.*e*. locus coeruleus) promotes NA activity, which in turn activates forebrain CRH activity and promotes EEG arousals [38–40]. One week of partially restricted sleep caused significant alterations in rat HPA axis response: decreased sensitivity of CRH and 5HT-1A receptors, while adrenal ACTH sensitivity was increased [37–41]. These alterations are similar to those seen in depression [37].

Open field test in the study by Tartar et al. [9] showed increased exploratory activity in 24h- sleep deprived and fragmented groups compared to exercise and cage controls. Unlike our results, their study suggested that observed alterations in behavioral pattern were the consequence of HPA stress response. Namely, the exercise control group, which received an equivalent amount of motion at treadmill, behaved in the open field test similarly as the cage control group. At the same time, this group showed a significant corticosterone elevation compared to the control group similar to the levels observed in sleep deprivation and sleep fragmentation groups. These results could be a consequence of prolonged, i.e. 24h-time spent on the treadmill track which could trigger stress response systems [40]. Moreover, according to McKanna et al. [17], 24h-sleep fragmentation is associated with significant alterations in REM sleep, which could be an additional confounding element. Taking into account the aforementioned findings, we chose to fragment animals for 6 hours, which is closer to their normal sleep time and reduces the chance of having stress as a confounding factor.

Higher prevalence of anxiety in females and hypogonadal men compared with otherwise healthy men has been observed in numerous studies [42,43]. Increased panic symptomatology during premenstruum was accompanied by abrupt decrease in progesterone levels, while these symptoms were alleviated during pregnancy [44], showing that gonadal hormones affect behavior. Progesterone has fundamental importance in the maintenance of steroid hormone homeostasis since it could be metabolized to several other steroids. Testosterone can undergo 5α-reduction which leads to the formation of dihydrotestosterone (DHT) or it can be aromatized to estradiol [45,46]. In addition, progesterone and testosterone serve as precursors for the synthesis of neurosteroids (dihydroprogesterone (DHP) and allopregnanolone or DHT and 3a-Diol respectively) [47].

Neurosteroid homeostasis is altered in depression and anxiety disorders. Antidepressants may act in part through restoring neurosteroid disbalance [48] since they are potent modulators of several neurotransmitter receptors such as GABA_A_, NMDA, AMPA, σ1 and glycine [49].

It has been shown that progesterone exerts anxiolytic and sedative effects in animals, as well as in men and women [50,51]. In this study, we showed that progesterone level was decreased in the group that underwent sleep fragmentation protocol (SF group, p<0.05). Present findings are consistent with previous studies [52–54] which suggested that progesterone modulates anxiety-like behavior. Specifically, administration of progesterone or its metabolites decreased anxiety-like behavior in male and female rodents [53–54]. Progesterone is much more than a female hormone because it can facilitate or even mimic the actions of testosterone in male-typical behaviors in species ranging from lizards to humans [55] and the effects of progesterone on anxiety are not sex-specific [56]. The effects of progesterone on anxiety levels are probably mediated by modulation of GABA_A_ receptor, whether directly or through its metabolite allopregnanolone [57]. This has been further proved by a study by Reddy et al. [50] which showed that progesterone receptor (PR) gene knockout mice exhibited greater anxiolytic response compared to the wild type, showing that most of these effects are probably mediated through allopregnanolone induced allosteric modulation of GABA_A_ [50]. Thus, sleep fragmentation may increase anxiety-like behavior by depletion of progesterone, as demonstrated in this study.

In this study, we showed that testosterone level was decreased in rats upon treadmill-mediated sleep fragmentation, compared to the corresponding control groups. Accordingly, our findings support the hypothesis that sleep fragmentation could contribute to low testosterone levels in patients with OSA. For a long period of time hypoxemia [58–60] and obesity [61] have been considered to be the main reasons for lower testosterone levels in patients with OSA, while sleep fragmentation has not been investigated as contributing factor until now. Intermittent hypoxia and sleep fragmentation occur simultaneously in OSA patients and their isolated effects could be assessed in experimental models. Recently, more complex models of OSA have been proposed [62], but the hormonal status has not been investigated.

Frye and Seliga [63] reported that testosterone supplementation in rats exhibited anxiolytic effect as assessed by increased exploratory behavior and open arm time in the elevated plus-maze test. Our results showed lower testosterone in rats exhibiting higher anxiety, which provides additional support for the role of androgens in reducing anxiety. That result is consistent with other findings confirming that testosterone administration to male rats increased open arm exploration [64]. Numerous studies have shown that castration of adult males is associated with increased anxiety-like behavior, which can be reversed by androgen replacement [63,65–68]. Prolonged testosterone treatment has been shown to decrease fear responses in a battery of tests involving unfamiliar stimuli [69, 70]. Testosterone is metabolized to form DHT, which can be further converted to 3α-diol and 3β-diol, neuroactive steroids with neuromodulatory activity [68]. Aikey et al. [71] suggested that anxiolytic effect of testosterone is mediated by 5α-reduced metabolites, which are powerful GABA_A_ agonists. Testosterone metabolites, androsterone and 3α-androstanediol, are weak androgens but potent modulators at GABA_A_/benzodiazepine receptor complexes (GBRs), whereas testosterone and DHT have low affinity for GBR [63,71] but act upon androgen receptors. Lower testosterone levels in the sleep-fragmented group could also be explained by predominance of REM sleep over SWS [72]. In addition, results of experimental studies showed that sleep deprived male rats had decreased testosterone concentrations [73]. These findings also support the hypothesis that the increase of anxiety-like behavior induced by sleep fragmentation, in our current study could be partially mediated by reduced testosterone levels.

In addition to being 5α-reduced, testosterone can also be aromatized to estradiol, which can modulate affective and cognitive behavior [74]. We demonstrated herein that estradiol levels were lower among experimental, sleep-fragmented rats compared to their controls. Proestrus female rats, with high circulating levels of estradiol and androgens, exhibited less anxious behavior relative to diestrus females (low levels of estradiol), as assessed by an increased number of open arm entries in the elevated plus-maze test, longer social interactions, and less freezing time in response to shock [75]. Our results are in agreement with the findings of Filova et al. [76] which have shown that estradiol administration in gonadectomized male rats exerts rapid anxiolytic effect on male rat behavior in the open field test, but not in other tests. Furthermore, Carrier et al. [77] demonstrated in previously gonadectomized male rats that anxiety-like behavior could be successfully reversed by supplementation with either testosterone or estradiol. However, estradiol independently has significant anxiolytic role [77]. Namely, chronic blockage of aromatase activity within the dentate gyrus showed a modulatory role of locally synthesized estradiol in organizing aspects of anxiolytic-like behavioral actions of testosterone. Since testosterone can also be aromatized to estradiol, and estradiol can alter affective behavior [75] and cognitive performance [74,78], we cannot rule out depletion of only one of them as a potential anxiety cause. That is also the reason for determining serum levels of both hormones (estradiol and testosterone).

In summary, our findings indicate that short-term sleep fragmentation enhances anxiety-related behavior in rats, as revealed by ethological tests. Sleep fragmentation demonstrated herein induced increase of anxiety-like behavior, which could be mediated by hormonal changes presented in the form of testosterone, estradiol and progesterone depletion.

These results shed more light on understanding the mechanisms of increased anxiety in patients with OSA, as well as on the contribution of sleep fragmentation to hormonal alterations.
